# Long COVID in Children and Adolescents

**DOI:** 10.3390/life12020285

**Published:** 2022-02-14

**Authors:** Valentina Fainardi, Aniello Meoli, Giulia Chiopris, Matteo Motta, Kaltra Skenderaj, Roberto Grandinetti, Andrea Bergomi, Francesco Antodaro, Stefano Zona, Susanna Esposito

**Affiliations:** 1 Pediatric Clinic, Department of Medicine and Surgery, University Hospital of Parma, 43126 Parma, Italy; valentina.fainardi@unipr.it (V.F.); aniello.meoli@gmail.com (A.M.); giulia.chiopris@gmail.com (G.C.); matteo.motta92@gmail.com (M.M.); kaltra.skenderaj@studenti.unipr.it (K.S.); robertograndinetti93@gmail.com (R.G.); 2 Local Health Agency of Modena, 41121 Modena, Italy; nao.bergomi@gmail.com (A.B.); francesco.antodaro@gmail.com (F.A.); 3 Primary Health Care Department, Local Health Agency of Modena, 41121 Modena, Italy; s.zona@ausl.mo.it

**Keywords:** COVID, long COVID, neuropsychiatric symptoms, quality of life, SARS-CoV-2

## Abstract

Severe acute respiratory syndrome Coronavirus 2 (SARS-CoV-2) has caused significant mortality and morbidity worldwide. In children, the acute SARS-CoV-2 infection is often asymptomatic or paucisymptomatic, and life-threatening complications are rare. Nevertheless, there are two long-term consequences of SARS-CoV-2 infection in children that raise concern: multisystem inflammatory syndrome in children (MIS-C) and long COVID. While the understanding and the experience regarding the acute phase of SARS-CoV-2 infection have remarkably increased over time, scientific and clinical research is still exploring the long-term effects of COVID-19. In children, data on long COVID are scant. Reports are conflicting regarding its prevalence, duration and impact on daily life. This narrative review explored the latest literature regarding long COVID-19 in the pediatric population. We showed that long COVID in children might be a relevant clinical problem. In most cases, the prognosis is good, but some children may develop long-term symptoms with a significant impact on their daily life. The paucity of studies on long COVID, including a control group of children not infected by SARS-CoV-2, prevents us from drawing firm conclusions. Whether the neuropsychiatric symptoms widely observed in children and adolescents with long COVID are the consequence of SARS-CoV-2 infection or are due to the tremendous stress resulting from the restrictions and the pandemics is still not clear. In both cases, psychological support can play a fundamental role in managing COVID pandemics in children. More knowledge is needed to share a standardized definition of the syndrome and improve its management and treatment.

## 1. Introduction

Severe acute respiratory syndrome Coronavirus 2 (SARS-CoV-2), the agent responsible for Coronavirus disease 2019 (COVID-19), has caused significant mortality and morbidity worldwide [1,2,3]. In children, the acute SARS-CoV-2 infection is often asymptomatic or paucisymptomatic, and life-threatening complications are rare [2]. Nevertheless, despite the low risk of COVID in the short term, there are two long-term consequences of SARS-CoV-2 infection in children that raise concern: multisystem inflammatory syndrome in children (MIS-C) and long COVID.

MIS-C is a rare phenomenon (to date, 783 cases worldwide have been reported) that occurs in children 2 to 6 weeks after the infection. It develops in less than 0.1% of children with COVID-19 (median age 8.6 years) and requires intensive care support in 68% of cases [3]. MIS-C is associated with marked immune activation and many potential mechanisms of immunopathogenesis. Children present with several multisystemic manifestations with those involving the gastrointestinal tract, skin, mucous membranes and the cardiovascular system reported as the most common. For some aspects, the clinical picture of MIS-C resembles that of Kawasaki disease (KD), especially in cases with shock, toxic shock syndrome and macrophage activation syndrome [4].

The second long-term consequence of SARS-CoV-2 infection is long COVID. Recent evidence shows that some of the symptoms that characterize the acute phase of SARS-CoV-2 infection may persist despite complete viral clearance both in adults and in children. According to the most recent definition by the National Institute for Health and Care Excellence (NICE), there are several entities determined by SARS-CoV-2 infection: (1) acute COVID-19, defined by signs and symptoms up to 4 weeks; (2) post-acute or ongoing symptomatic COVID-19, when symptoms persist from 4 to 12 weeks; (3) post-COVID-19 syndrome, defined by signs and symptoms that continue for more than 12 weeks after onset of acute symptoms. Long COVID includes both post-acute COVID-19 and post-COVID-19 syndrome [5]. Long COVID is largely studied in adults, mainly those hospitalized [6,7,8], but prevalence, clinical manifestations and treatment are still a matter of study. In a recent systematic review including 250,351 survivors of COVID-19, more than half had at least one residual symptom up to 6 months or more from the infection [7]. In children, data on long COVID are scant. Reports are conflicting regarding its prevalence, duration and impact on daily life. This narrative review aims to explore the latest literature regarding long COVID-19 in the pediatric population.

## 2. Epidemiology of Long COVID in Children

Since COVID-19 prevalence varies between countries, an accurate prediction of children who will progress to long COVID is difficult to make. Moreover, studies on long COVID are mostly based on self or parent-reported symptoms without an objective clinical evaluation or without a previous proven SARS-CoV-2 infection [9,10,11,12,13,14,15,16,17].

The prevalence of long COVID-19 in children varies remarkably depending on the studies, ranging from 4% to 66% (Table 1) [18,19,20,21,22,23,24].

Studies are very heterogeneous, differing in sample size, the median age of the included population, duration and modalities of follow-up. In particular, 6 out of the 14 studies reporting on long COVID prevalence conducted a face-to-face assessment in post-COVID-19 dedicated clinics, whereas the remaining studies collected data via online platforms or phone interviews.

One of the first reports on long-COVID in children was a case report of five Swedish children (median age 12 yrs) who had symptoms lasting 6-8 months after the acute infection. None of these patients required hospitalization during the acute phase, but one patient with associated comorbidities required admission after three months because of peri-myocarditis [9].

An Australian study followed 171 children in a dedicated post-COVID-19 clinic: 8% reported post-COVID symptoms up to 8 weeks, and all of them were symptomatic at the time of infection [10].

An Italian cross-sectional study interviewed the parents of 129 children reporting that 66.6% had at least one persisting symptom after 60–120 days from the initial diagnosis, 27.1% after 120 days or more and 42.6% after three months. Surprisingly, even asymptomatic or paucisymptomatic cases in the acute phase of the infection developed persisting symptoms [11].

A prospective cohort Russian study collected data on 518 children (median age 10.4 yrs, 44.7% with associated comorbidities) affected by COVID-19, and 24.7% of them had at least one persisting symptom after the acute phase with some of the symptoms lasting up to 6–7 months [12].

In Israel, a prospective cohort study collected data from 90 children in a multidisciplinary center for long-COVID. The median number of chronic symptoms was four (1–14), and in almost 60% of patients, symptoms were associated with functional impairment after one to seven months after illness onset [13].

In England and Wales, the VirusWatch study collected data from 4678 children, including children affected by COVID-19 (*n* = 174) and healthy controls (*n* = 4504). The monthly survey showed that symptoms lasting for more than four weeks were reported by 4.6% of children with a history of SARS-CoV-2 (8/174) and by 1.7% of controls (72/4504) [14].

Data from 510 children living in the United Kingdom and the United States were collected using an online platform created by an association of parents called LongCOVIDKids. Parents had to fill out an online survey regarding the status of the child before the COVID infection, the characteristic of the acute phase and the persistence of chronic symptoms. In 58.2% of the cases, COVID-19 was confirmed by a microbiological test; in 41%, the infection was suspected only clinically. Only children with symptoms lasting for more than four weeks were included. The median duration of symptoms was 8.2 months; 25.3% had constant long-COVID symptoms, 49.4% had fluctuating symptoms and 19% suffered from returning symptoms after a period of prolonged wellness [15].

Molteni et al. acquired data from children who tested positive for SARS-CoV-2 (*n* = 1734) and matched negative controls through a mobile application launched by ZOE Global. Parents could report their children status using a questionnaire. Data were collected between March 2020 and February 2021. Of the children who tested positive, the median illness duration was 6 days (vs. 3 days in the matched controls), 4.4% had symptoms lasting more than four weeks and at least for 28 days, with a mean number of symptoms of six in the first week and two at day 28. Only 1,8% was still symptomatic after day 56. Among the matched negative controls, less than 1% was symptomatic for more than 28 days [16]. This prevalence in the UK was recently confirmed by the Office for National Statistics (ONS) that reported the prevalence of symptoms 4–8 weeks post-infection as follows: 3.3% in primary-school-aged children and 4.6% in secondary-school aged children, with a matched control group symptom prevalence of 3.6% in primary-school-aged children and 2.9% in secondary-school aged children [17].

One of the latest studies on long-COVID was conducted at the Children’s Clinical University Hospital in Latvia. This cohort study enrolled 236 pediatric COVID-19 patients and 142 comparison group patients to identify the long-term consequences of SARS-CoV-2 infection 1–6 months after acute infection. After a median follow-up time of 73.5 days, almost 70% of participants had at least one persisting symptom and more than the 50% had two or more [18].

## 3. Pathophysiology and Immunological Aspects

The underlying mechanisms causing the various clinical spectrum of long COVID are still unknown. The angiotensin-converting enzyme 2 (ACE2) receptor is the gateway that SARS-CoV-2 uses to invade the cells of many organs (lungs, heart, brain, oral and nasal mucosa, gastrointestinal tract, pancreas, liver, spleen, kidney, blood vessel endothelium and others) causing acute damage [25].

The wide ACE2 receptor epithelial expression, as already well documented by pathology studies during the 2004 epidemic caused by SARS-CoV, could explain the variety of symptoms that also characterize long COVID [26]. In patients where the injury induced by the initial viral infection results in persistent symptoms, there might be a combination of the following mechanisms: chronic tissue damage, the persistence of the virus in the organism and dysregulation of the immune system with consequent autoimmunity and chronic inflammation [27].

The altered immunological response seems to play a predominant role. A recent study (not yet peer-reviewed) described the immunological differences between children completely recovered from the acute infection and children with post-acute sequelae (defined as PASC) [28].

This study provided the first objective laboratory evidence of the existence of PASC in children, previously described in adults. Children with PASC (*n* = 12 of whom three had pre-existing comorbidities, mean age 10.3 ± 4.5 yrs) showed higher levels of plasmablasts, IgD-CD27+ memory and switched IgM-IgD-B cells. On the other hand, the group of children completely recovered (*n* = 17 of whom three had pre-existing conditions, mean age 7.7 ± 5.5 yrs) had a higher number of naive and unswitched IgM+IgD+ and IgM+CD27-CD38dim B cell subsets, indicating the restoration of B cell homeostasis. Interleukin (IL)-6 and IL-1, mediators of inflammatory responses and auto-immune processes, were elevated in PASC patients and consistently higher than in recovered children suggesting the predominant role of the innate immune system.

According to the model proposed above, in the alveoli, after the initial damage to the blood–lung barrier, chronic inflammation with continuous production of pro-inflammatory cytokines and reactive oxygen species (ROS) may occur, followed by their release into surrounding tissues and bloodstream. This process can lead to the activation of complement, platelets and platelet–leukocyte interactions with further release of pro-inflammatory cytokines and impaired coagulation [27]. Abnormalities in coagulation can increase the risk of microthrombosis in multiple organs but especially in alveolar capillaries (given the extremely rich expression of ACE2 receptor in lungs and blood vessels endothelium) with consequent hypoxemia, tissue hypoxia and eventually dyspnea, as described in the adult population [29,30]. Therefore, the prolonged hyperinflammatory and hypercoagulable state can increase the risk of thrombosis, which may partly explain the common respiratory symptoms complained by patients with long COVID.

All these aspects were well exemplified by a case report on a 14-year-old girl initially diagnosed with mild COVID-19 infection in October 2020. Due to persisting symptoms, she was hospitalized 7 months later. Laboratory investigations were suggestive of a pro-inflammatory state (high level of IL-6, IL-1, TNF-α, unusual B-cell and regulatory T-cell patterns, low concentrations of CD27+ memory B lymphocytes), high concentrations of IgA and IgG anti-SARS-CoV-2 antibodies were found and cardiopulmonary exercise testing (CPET) showed mild pulmonary hypertension.

The recent similar approach used in adults with long-term COVID-19 sequelae and the finding of pulmonary hypertension, guided the investigators to perform pulmonary scintigraphy and ventilation–perfusion single-photon emission computed tomography (V/Q SPECT), the gold-standard screening test in the assessment of chronic thromboembolism. Whereas planar scintigraphy did not show areas with decreased radiopharmaceutical uptake in both lungs, V/Q SPECT showed significant perfusion defect in the apical segment of the right upper lobe, with no correspondent parenchymal alterations on co-registered CT slices. These findings could be explained by microvascular and endothelial damage, and this was the first reported evidence of organ dysfunction in an adolescent with PASC [31,32].

Interestingly, in a small percentage of adult patients, a pro-fibrotic state can be observed: the released cytokines, especially IL-6, along with activation of fibroblasts following acute damage, can lead to deposition of collagen and fibronectin and lead to long-term fibrotic abnormalities [33,34]. Further studies are needed to better characterize the pro-fibrotic state and the underlying mechanisms in children, along with a better comprehension of the sequelae [35].

As seen for the lung, in the heart, the endothelial damage and the microthrombosis can lead to abnormalities in coagulation pathways. Fibrotic changes are possibly favored by prolonged immune activation and accompanied by an increase in cardiac fibromyoblasts. The chronic inflammation of cardiomyocytes can result in myositis and cause cardiomyocytes death due to an intense local immune response [27]. Furthermore, specific cardiovascular dysfunction of the afferent autonomic nervous system (caused by the combination of infection, autonomic nervous system pro-inflammatory response and autoimmunity) can lead to complications such as postural orthostatic tachycardia syndrome (POTS) [36].

In the central nervous system, the infection may occur by haematogenic or neuronal retrograde routes; even the blood–brain barrier may be affected by the infection, causing chronic neuroinflammation [27]. The long-term immune response can activate glial cells, which chronically damage neurons, and the abnormalities of coagulation may lead to an increased risk of thrombotic events. The blood–brain barrier dysregulation can then allow cytokines and leukocytes to infiltrate the brain parenchyma, and the effect of chronic neuroinflammation in the brainstem could be the basis of autonomic dysfunction [37]. All these mechanisms can lead to the multiple neurological manifestations (such as cognitive impairment) ascertained in patients with long COVID.

**Figure 1:**
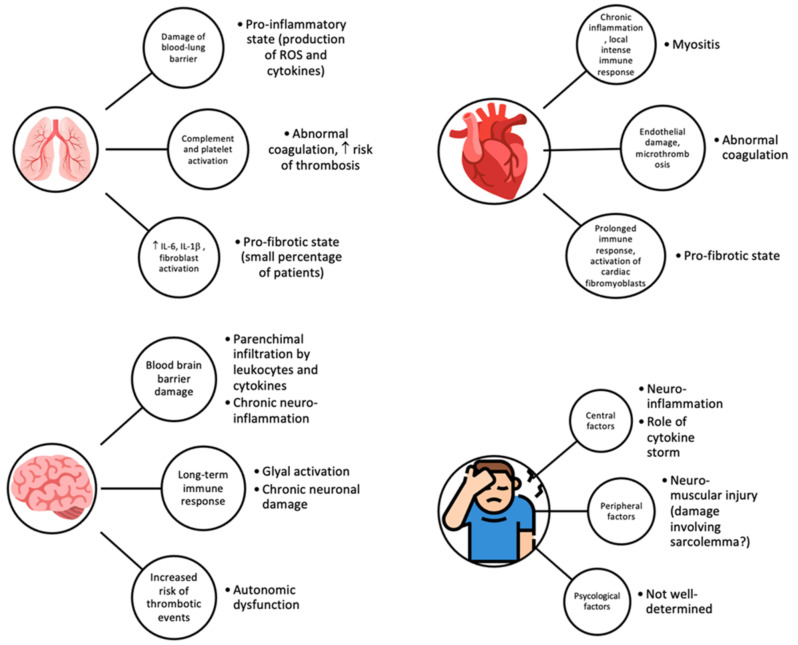
Proposed organ-specific mechanisms of injury of long COVID.

The gastroenteric tract is a frequent target of SARS-CoV2 infection, given the elevated expression of ACE2 receptors along the mucosa but also in other organs (pancreas, liver, spleen). Although the exact mechanism is still not fully understood, it is highly probable that the direct attack of SARS-CoV-2 is relevant in causing gastrointestinal symptoms either in COVID-19 or in long COVID, always referring to the proposed model in Figure 1 [27]. The pathogenetic steps leading to the persisting gastrointestinal symptoms may be diffuse tissue damage of the bowel leading to enterocytes desquamation, edema and small bowel dilation, immune dysregulation with lymphocytes infiltration, microthrombosis and necrosis [38,39].

Finally, one of the most frequently described symptoms in long COVID is fatigue. The most likely hypothesis involves the simultaneous presence of central, peripheral and psychological factors [26]. The already cited chronic neuroinflammation, along with neuromuscular mechanisms of injury (such as damages involving sarcolemma), may result in long-term fatigue, but non-well determined psychological and social factors might play a part.

Proposed mechanisms implicated in manifestations of long COVID in different organs are shown in Figure 1.

Future research might focus more on bio-humoral aspects, especially on cytokine storm, which seems to play a significant role especially when compared to similar problems encountered in past epidemics [40]. However, as we have seen, studies were carried out mainly in adults and data on pediatric patients are still lacking. Many mechanisms appear to be similar in adults and children, but targeted studies are needed to better understand the pathophysiology of long COVID in children [41].

## 4. Clinical Manifestations

The clinical manifestations of long-COVID are highly variable in symptoms, intensity and duration. The signs and symptoms resumed in Table 2, can occur both alone and in combination, can be transient or intermittent, can change over time or remain constant. The range of clinical manifestations reported in the different studies is quite wide, highlighting the heterogeneity in clinical presentation.

Although long COVID manifestations seem to be more common in children with symptomatic or severe acute SARS-CoV-2 infection, they are also described in asymptomatic or paucisymptomatic patients [15].

The few studies conducted in children demonstrated the same symptoms of long-COVID reported in the adult population.

As in adults [42], the most common symptom described in children was fatigue, reported in up to 87% of patients [9,10,11,12,15,18,20]. Some authors [20] highlighted that fatigue could be determined by the limitations in daily activities due to school unattendance. However, this datum is difficult to verify in the absence of a control group.

In a five-patient case series on long-COVID, Ludvigsson et al. reported that all children complained of fatigue for 6–8 months, and none were able to go back to school full time [9].

In an Italian study, parents reported that most children affected by COVID-19 showed a certain impairment in physical activity due to fatigue: after 4 weeks from the acute infection, 30.2% experienced a worsening of the symptoms in case of physical activity and 21.2% could not practice any. Only 11.8% of children returned to previous levels of physical activity [15]. However, as mentioned above, restrictive measures might have contributed to these results.

To date, six studies compared children infected with COVID-19 and a control group [14,16,18,19,21,23] and four [14,16,18,23] reported a higher prevalence of symptoms in children affected by COVID, suggesting long COVID syndrome.

The cohort study conducted by Roge et al. at the Children’s Clinical University Hospital in Latvia compared children previously affected by SARS-CoV-2 (19 children) and children with other non-SARS-CoV-2 community-acquired infections. Overall, 70% of patients complained of at least one persistent symptom and 54% of multiple persistent symptoms. The persistence of at least one symptom [fatigue (25.2%), irritability (24.3%), mood changes (23.3%), headaches (16.9%), rhinorrhea (16.1%), coughing (14.4%), anosmia/dysgeusia (12.3%)] was more frequent in patients with COVID-19 infection (70% vs. 24.8%, *p*-value < 0.001). Interestingly, school-age children and adolescents were mostly affected by cognitive and neurological disorders while infants and preschoolers by respiratory problems [18].

Stephenson et al. [23], in The Children and Young People with long COVID (Clock) study, interviewed with a questionnaire 3065 children aged 11–17 years tested positive for SARS-CoV-2 and a total of 3793 age-, sex- and geographicaly matched SARS-CoV-2-negative controls. Three months after the test, the most common neuropsychiatric symptom was a headache with a higher prevalence in the group of the subjects who tested positive for COVID (23.2% vs. 14.2%); no difference was reported in mental health, wellbeing, quality of life and fatigue.

Blankenburg et al. [19] enrolled a total of 1560 students (median age, 15 yrs), of which 1365 (88%) were seronegative for COVID-19 and 188 (12%) seropositive. The children were asked to complete a validated questionnaire with 12 questions on the occurrence and frequency of pain and relevant neurocognitive or mood symptoms within the last seven days. Each symptom was documented in about 35% of the students without difference between the two groups. Unhappiness (98.7%), tenseness (86.4%), listlessness (80.7%) and difficulties in concentrating (79.3%) were the most common reported issues.

Overall, neuropsychiatric symptoms appear to be very common in children after the infection with COVID-19.

In the first study regarding long COVID in children [11], insomnia (18.6%), headache (10.1%) and concentration difficulties (10.1%) were among the most frequently reported symptoms both in symptomatic and asymptomatic children during the acute phase of the infection, and these persisted in 42.6% of cases 120 days after the diagnosis.

The Swedish five-patient case series highlighted the presence of neuropsychiatric symptoms of long COVID up to 8 months since the infection. Headaches, difficulties concentrating and dizziness were reported in four patients, memory loss and depression in three patients and sleep disorders in two patients. In addition, two out of five children had seen, or were scheduled to see, a psychologist [9].

In an Italian study, Buonsenso et al. performed a comprehensive assessment of parents’ perspectives derived from an online survey in order to establish long-term physical and mental health outcomes in 510 children with persisting symptoms after acute SARS-CoV-2 infection. The authors focused on reported symptoms, their dynamics and the overall changes in children’s activities and mental health. Overall, the mean duration of symptoms was 8.2 months with a significant prevalence of neuropsychiatric symptoms such as headache (78.6%), lack of concentration (60.6%), unexplained irritability (51.4%) and dizziness (48%), difficulty remembering information (45.9%), difficulty in doing everyday tasks (40%), difficulty processing information (32.7%) and short-term memory issues (32.7%). In addition, most parents reported changes in energy levels (83.3%), mood (58.8%), sleep (56.3%) and appetite (49.6%). In 25.3% of cases, children suffered from persistent symptoms; in 49.4%, there was a period of apparent recovery after the infection, and then symptoms returned, and in 19%, symptoms appeared after a prolonged period of wellbeing [15].

Further characterization of symptoms belonging to the neuropsychiatric sphere was described in children after COVID infection by a survey conducted by Brackel et al. among Dutch pediatricians. Eighty-nine children aged 2–18 years with various disorders ascribed to long COVID were identified. Many patients reported cognitive dysfunctions (45% concentrating difficulties, 13% memory loss, 2% brain fog) and headaches (38%). Furthermore, 48% and 36% of children reported mild or severe limitations, respectively, in daily routine and school attendance. Of note, 16% of subjects were seen by a psychologist [20].

Osmanov et al. conducted one study in a cohort of 518 subjects previously hospitalized for COVID-19, assessing symptoms prevalence and duration of long COVID using the International Severe Acute Respiratory and Emerging Infection Consortium (ISARIC) COVID-19 Paediatric follow-up questionnaire. After fatigue (10.6%), sleep disturbances were the second most frequent symptom (7.2%), followed by sensory problems (6.2%). Regarding the decline of symptoms over time, smell disturbances declined faster than headache and sleep problems. This finding may be attributable to a psychological mechanism rather than to an effect of the viral infection. Moreover, parents thought that COVID-19 illness, and not to the pandemic in general, was the cause of reduced appetite (4.5%), reduced (3.5%) or augmented (2%) sleeping, reduced physical activity (4.7%) and reduced emotions (4.3%) [12].

In another study conducted in Iran on 58 children and adolescents previously admitted to the hospital with a COVID-19 diagnosis, sleep difficulty (5%) and headaches (5%) were the most frequent neuropsychiatric symptoms 3 months after discharge [43].

However, the variability of symptoms in patients with long COVID-19 is wide. Miller et al., in their household cohort study on 4678 children, showed that long COVID could affect all systems: eye–nose–throat symptoms (22.5%) were the most common after fatigue (27.5%), followed by symptoms affecting the respiratory (21.2%), neurological (16.2%), dermatological (15%), gastrointestinal (13.8%), cardiovascular (11.2%), psychiatric (10%) and muscular (8.8%) system [14].

In a recent systematic review on long COVID symptoms, 14 studies heterogeneous for design, inclusion criteria, outcomes and follow-up were included. The prevalence of long COVID symptoms in the included children (*n* = 19.426) ranged from 4 to 66%. The most common symptoms were headache (3–80%), fatigue (3–87%), concentration difficulties (2–81%), sleep disturbance (2–63%), abdominal pain (1–76%) and myalgia or arthralgia (1–61). In the majority of the studies, all symptoms did not persist longer than 12 weeks. The wide variability shown in symptoms ‘prevalence might be caused by some limitations of the included studies such as the different ages of the subjects, the lack of a unique definition of long COVID, the absence in some cases of objective clinical evaluations, the low response rates to questionnaires in asymptomatic or paucisymptomatic subjects and the absence of control groups [44].

Further studies with more subjects and negative control groups are needed to establish whether the features associated with long COVID are related to the viral infection or to the effects of lockdown, school closures and social isolation [23].

## 5. Risk Factors for Long COVID

Risk factors for long COVID in children were investigated in a few studies.

Osmanov et al. reported that older age was significantly associated with persistent symptoms, with children above 6 years of age being at higher risk [12]. Molteni et al. described illness duration in a large cohort of symptomatic school-aged children tested for SARS-CoV-2 and reached similar conclusions reporting that children older than 12 years were more likely to manifest symptoms over the following 28 days (5.1% vs. 3.1%, *p* = 0.046); this evidence was not confirmed over the following 56 days (2% vs. 1.3%, *p* = 0.52) [16]. Other studies showed a positive correlation between increasing age and long COVID symptoms [13,14,19,23].

Female sex was described as another risk factor for long COVID by three recent studies [14,19,23].

Allergic disease has also been described as a possible risk factor for long COVID in children [12]. Similarly, studies in adults reported that asthma was associated with a higher risk of developing long COVID [6,45]. This can be explained by mast cell activation syndrome and the Th2 based immunological response, mechanisms overexpressed in patients with allergic disease.

## 6. Long Term Consequences of Long COVID in Pediatric Age

Data on long-term pulmonary impairment in children with previous SARS-CoV-2 infection are conflicting. Denina et al. observed that patients with abnormalities at lung ultrasound during hospitalization showed complete normalization within 5 weeks [46].

On the other hand, Ashkenazi-Hoffnung et al. showed mild radiologic and spirometric changes in more than half of their patients in follow-up for long COVID [13].

Further studies might be useful in order to define the risk of persistent impaired respiratory function after SARS-CoV-2 infection in children.

## 7. Monitoring and Follow-Up

Data indicate that long COVID in children is a real problem. This means that all children with suspected or documented COVID-19 infection and their parents, even if asymptomatic at the time of diagnosis, must be informed about the possible persistence of symptoms for more than 4 weeks and about the possible reappearance after the acute phase with the same or different characteristics. Adolescents, females and children with underlying diseases, including mental health, should be particularly warned because they are at increased risk of long COVID. However, present knowledge of the characteristics of pediatric long COVID is largely incomplete, and this makes it difficult to indicate which is the most rational approach to this condition in children.

**Figure 2:**
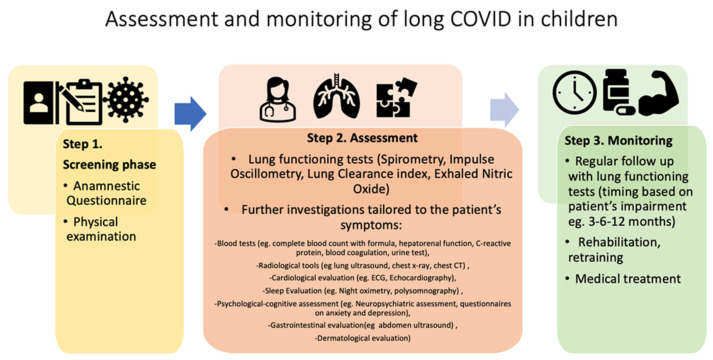
Screening, assessment and monitoring of children infected by COVID-19.

Considering the multi-organ involvement seen in long COVID, a multidisciplinary team is mandatory in the follow-up of these patients. In Figure 2, we propose a stepwise approach for screening, assessing and monitoring children infected by COVID 19.

According to the recommendations of NICE, the first step can be a questionnaire to determine the acute phase of the disease and assess the most common symptoms of long COVID [5]. Questionnaires such as the COVID-19 Yorkshire Rehabilitation Screen (C19-YRS) [47] or the questionnaire developed by the Swiss COVID Study Group and the Swiss Society for Pulmonology (SSP) were used to address the diagnosis and treatment of pulmonary long COVID in adult patients [48]. Conversely, no specific and standardized questionnaires are available for the pediatric population.

Studies demonstrated that immediately after the acute phase of COVID-19 infection (4–12 weeks), a very small number of children presented symptoms; mostly physical symptoms. During this period, all children who were previously infected with COVID-19 were examined by the general pediatrician and further investigations were prescribed on an individual basis.

Depending on their clinical history and physical examination, children can enter a second phase that includes pulmonary assessment. In adults, pulmonary assessment is recommended in discharged patients with persistent respiratory symptoms [49]. The British Thoracic Society recommends chest X rays 3 months after discharge for all adult patients admitted to the hospital with COVID-19 infection, particularly in those with a history of moderate or severe disease; patients with persistent symptoms or radiological abnormalities are offered further clinical referrals and investigations [50]. In children, pulmonary assessment can include various lung function tests and further investigations depending on reported symptoms and clinical history; some patients may require medical treatment such as bronchodilators or inhaled steroids.

A single pediatric study conducted in Israel on 90 children with persistent symptoms found mild radiologic and spirometric changes in more than half of the patients. Conversely, none of the children exhibited abnormal findings on echocardiography, raising the question of whether this investigation is necessary for children with normal echocardiography and no story of MIS-C [13].

Step 3 includes the monitoring phase with visits and lung function measurements at 3, 6 and 12 months, depending on the patient’s impairment.

In conclusion, we think that primary care pediatricians should visit all subjects with a suspected or a proven diagnosis of SARS-CoV-2 infection after 4 weeks to check for the presence of symptoms of a previously unknown disease. In any case, a further check-up by the primary care pediatrician should be scheduled 3 months after the diagnosis of SARS-CoV-2 infection to confirm normality or to address emerging problems. The subsequent approach to the individual case will have to be evaluated on the basis of the present symptoms.

## 8. Conclusions

While the understanding and the experience regarding the acute phase of SARS-CoV-2 infection have remarkably increased over time, scientific and clinical research is still exploring the long-term effects of COVID-19, particularly in children. Long COVID in children might be a relevant clinical problem. In most cases, the prognosis is good, but some children may develop long-term symptoms with a significant impact on their daily life. The paucity of studies on long COVID, including a control group of children not infected by SARS-CoV-2, prevents us from drawing firm conclusions. Whether the neuropsychiatric symptoms widely observed in children and adolescents with long COVID are the consequence of SARS-CoV-2 infection or are due to the tremendous stress resulting from the restrictions and the pandemics is still not clear. In both cases, psychological support can play a fundamental role in managing COVID pandemics in children.

More knowledge is needed to: (1) recognize the true prevalence of long COVID in children; (2) better understand phenotype and pathogenesis of this disease; (3) share a standardized definition of the syndrome; (4) improve management and treatment; (5) analyze the impact of different viral variants on long COVID in pediatrics. In the meantime, vaccination in children and adolescents appears crucial to protect them from the long-term consequences of SARS-CoV-2 infection. Further studies could evaluate the impact of COVID vaccination on long COVID prevalence.

## Figures and Tables

**Table 1:** Long COVID studies conducted on the pediatric population with particular reference to its prevalence; m = months, w = weeks, d = days.

Study Reference	Study Design	Country	Number of Subjects	Controls	COVID-19 Laboratory Confirmed	Subjects with Associated Comorbidities (%)	Symptomatic at Time of Infection (%)	Need of Hospitalisation during Acute Phase *(%)*	Subjects with Long COVID (*%)*	Age (years), Median or Mean	Median Follow-Up
Ashkenazi-Hoffnung et al., 2021 [13]	Prospective cohort study	Israel	90	No	Yes	31.1	96.7	12.2	100	12.5	112 d
Blankenburg et al., 2021 [19]	Cross-sectional	Germany	1560 (188 seropositive)	Yes	Yes	-	-	-	-	15 (14–16)	-
Brackel et al., 2021 [20]	Cross-sectional	Netherlands	89	No	No	-	-	-	100	13 (9–15)	-
Buonsenso et al., 2021 [11]	Cross-sectional	Italy	129	No	No	-	74.4	4.7	58.2	11 ± 4.4	162.5 ± 113.7 d
Buonsenso et al., 2021 [15]	Cross-sectional	UK, USA	510	No	Yes	56.3	87.8	4.3	93.7	10.3 ± 3.8	-
Miller et al., 2021 [14]	Prospective cohort study	UK	4504 (175 positive)	Yes	Yes	-	-	-	4.6	0–17	-
Molteni et al., 2021 [16]	Prospective cohort study	UK	3113 (1734 positive)	Yes	Yes	13.1	-	-	4.4	5–17	4 w
Osmanov et al., 2021 [12]	Prospective cohort study	Russia	518	No	Yes	44.7	-	-	24.7	10.4 (3–15)	268 d
Radtke et al., 2021 [21]	Prospective cohort study	Switzerland	1355 (109 positive)	Yes	Yes	-	-	-	4	11	12 w
Roge et al., 2021 [18]	Cohort-study	Latvia	236	No	Yes	22	93	-	64.4	10	73.5 d
Say et al., 2021 [10]	Prospective cohort study	Australia	171	No	Yes	14	64	8	8	3.7 ± 3.5	3–6 m
Smane et al., 2020 [22]	Retrospective cohort study	Latvia	30	No	Yes	23	83	16.6	30	9.2 ± 5.2	101 d
Stephenson et al., 2021 [23]	Prospective cohort study	UK	6804 (3065 positive)	Yes	Yes	-	-	-	66.5	11–17	15 w
Sterky et al., 2021 [24]	Prospective cohort study	Sweden	55	No	Yes	35	-	-	22	0–18	219 d

**Table 2:** Prevalence of clinical manifestations in children with long COVID.

Cardiovascular	Neurological/Neuropsychiatric
Chest tightness or pain (1–31%)	Brain fog
Palpitations (4–18%)	Concentration difficulties (2–81%)
**Dermatological**	Sleep disturbance (2–63%)
Skin rashes (2–52%)	Dizziness (3–20%)
**Gastrointestinal**	Irritability and mood changes (5–24%)
Stomachache (5–70%)	Headache (3–80%)
Abdominal pain (1–76%)	Memory loss
Diarrhea (2–24%)	Smell disorder (12–70%)
Vomiting (2–24%)	Taste disorder (20–70%)
**General**	Nocturnal sweating
Fatigue (3–87%)	**Respiratory**
Persistent fever (2–40%)	Cough (1–30%)
Loss of appetite or weight (2–50%)	Dyspnea (40–50%)
**Muscular**	**Upper Airway**
Myalgia or arthralgia (1–61%)	Nasal congestion or rhinorrhea (1–12%)
	Sore throat (4–70%)
